# *In vivo *quantification of formulated and chemically modified small interfering RNA by heating-in-Triton quantitative reverse transcription polymerase chain reaction (HIT qRT-PCR)

**DOI:** 10.1186/1758-907X-1-16

**Published:** 2010-08-23

**Authors:** Yosef Landesman, Nenad Svrzikapa, Armand Cognetta, Xuemei Zhang, Brian R Bettencourt, Satya Kuchimanchi, Keri Dufault, Sarfraz Shaikh, Maple Gioia, Akin Akinc, Renta Hutabarat, Rachel Meyers

**Affiliations:** 1 Alnylam Pharmaceuticals, Cambridge, MA, USA

## Abstract

**Background:**

While increasing numbers of small interfering RNA (siRNA) therapeutics enter into clinical trials, the quantification of siRNA from clinical samples for pharmacokinetic studies remains a challenge. This challenge is even more acute for the quantification of chemically modified and formulated siRNAs such as those typically required for systemic delivery.

**Results:**

Here, we describe a novel method, heating-in-Triton quantitative reverse transcription PCR (HIT qRT-PCR) that improves upon the stem-loop RT-PCR technique for the detection of formulated and chemically modified siRNAs from plasma and tissue. The broad dynamic range of this assay spans five orders of magnitude and can detect as little as 70 pg duplex in 1 g of liver or in 1 ml of plasma. We have used this assay to quantify intravenously administrated siRNA in rodents and have reliably correlated target reduction with tissue drug concentrations. We were able to detect siRNA in rat liver for at least 10 days post injection and determined that for a modified factor VII (FVII) siRNA, on average, approximately 500 siRNA molecules per cell are required to achieve a 50% target reduction.

**Conclusions:**

HIT qRT-PCR is a novel approach that simplifies the *in vivo *quantification of siRNA and provides a highly sensitive and reproducible tool to measure the silencing efficiency of chemically modified and formulated siRNAs.

## Background

Several small interfering RNA (siRNA)-based therapeutics are currently in various phases of preclinical and clinical development [1-3]. There is an unmet need to develop sensitive methods to detect and quantify siRNAs in cells and tissues. Therapeutic siRNAs for systemic delivery are typically chemically modified and formulated. Chemical modifications of the sugar-phosphate backbone stabilise siRNA duplexes by enhancing their nuclease resistance and increasing their specificity by reducing off-target effects [4]. Of all delivery systems described, the formulation of siRNAs within cationic lipid nanoparticles (LNPs) is the most validated method for delivery to liver and possibly to other organs [5]. While chemical modifications contribute to increased siRNA stabilisation and specificity, they can significantly increase duplex melting temperature. Moreover, although formulations enhance siRNA tissue delivery and cellular uptake, they can inhibit siRNA release and detection. Currently used siRNA quantification methods include visualisation of radiolabelled siRNAs, hybridisation-based assays with labelled probes using enzyme-linked immunosorbent assays (ELISAs; specifically, hybridisation ELISA), methods based on high performance liquid chromatography (HPLC), capillary gel electrophoresis (CGE) and liquid chromatography mass spectrometry (LC-MS) alone or in combination with hybridisation-based assays (reviewed by Tremblay and Oldfield [6]). The most sensitive of these bioanalytical methods is the hybridisation ELISA with lower limit of quantification (LLOQ) of approximately 1 ng/ml of a 20-mer oligonucleotide in plasma and approximately 1 ng/g in liver [7]. However, these assays are laborious and have limited sensitivity. A more sensitive assay that is simple and fast to perform and that allows the quantification of therapeutic siRNAs from tissue and plasma is greatly needed.

Recently, Chen *et al*. developed a stem-loop quantitative reverse transcription PCR (qRT-PCR) method for the quantification of microRNAs (miRs) from tissue culture cells [8]. This assay is particularly appealing because of its high sensitivity, selectivity, and broad dynamic range for the detection of short RNA single strands characteristic of mature miRs. However, when applied to the detection of siRNA duplexes in liver, the stem-loop RT-PCR assay demonstrated a poor dynamic range and suboptimal amplification, suggesting inefficiencies in the RT and PCR steps [9,10]. In addition, amplification efficiency was lower for chemically modified versus unmodified siRNAs [9]. Such differences may result from the double-stranded nature of siRNAs, their chemical modifications and formulations.

Here, we describe the heating-in-Triton (HIT) method that, when used in combination with the stem-loop qRT-PCR technique, allows robust siRNA quantification from cell lines, tissues and plasma. Using this assay we measured unmodified and chemically modified siRNAs, either as unformulated (naked) siRNAs, or as formulated duplexes within LNPs [11,12]. Importantly, this assay is strand specific and thus requires complete denaturation of the sense and antisense strands. The assay is highly specific and sensitive, with a broad dynamic range of approximately five orders of magnitude that allows the detection of as little as 70 pg siRNA in 1 g of tissue or 1 ml of plasma. With simple and rapid sample processing, high reproducibility and applicability independent of the chemical modification or delivery strategy employed, this assay is robust and easily adapted for high throughput bioanalytical quantification of siRNA *in vivo*.

## Results

### siRNA quantification requires duplex liberation from the formulation followed by strand separation

**Figure 1:**
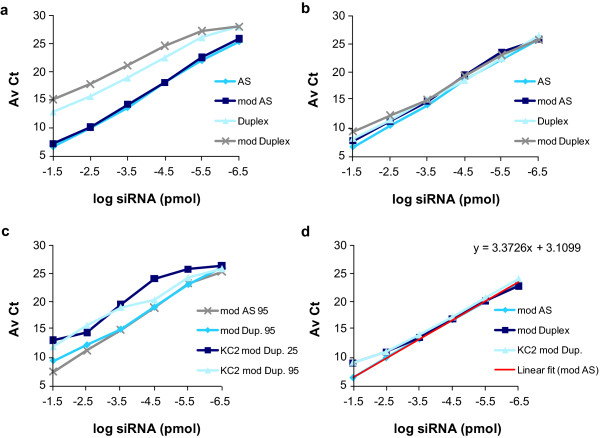
**Efficient small interfering RNA (siRNA) quantification requires release from the formulation and strand separation**. Amplification curves of unmodified (antisense (AS)), modified AS (mod AS), duplex (Duplex), modified duplex (mod Duplex), and lipid nanoparticle (LNP)-KC2-formulated modified duplex (KC2 mod Dup) siRNAs. siRNAs were diluted as indicated either in water or in 0.25% Triton. Then were treated as indicated either at room temperature (25°C) or heated to 95°C for 10 min and added directly into reverse transcription reactions: **(a) **water; 25°C, **(b) **water, 95°C, **(c) **water, 95°C for mod AS, mod Dup and for KC2-mod Dup, both in 25°C and 95°C, and **(d) **0.25% Triton-phosphate-buffered saline (PBS); 95°C. The linear regression of the amplification curve for chemically modified AS siRNA is shown (inear fit (mod AS)). The average cycle threshold (AvCt) values (n = 3) indicate signals in single PCR reactions containing siRNA equivalent concentrations ranging between 3.35 × 10^-2 ^to 3.35 × 10^-7 ^pmol.

We evaluated the amplification of two naked siRNA duplexes and their antisense single strands by qRT-PCR using the stem-loop method [8]. Both siRNAs target rat factor VII (FVII) and have identical sequences, but differ in backbone chemical modifications that increase the melting temperature of the modified duplex: AD1661 (modified duplex) is identical to AD1596 (duplex) except that it contains 2'-fluoro-chemically modified nucleotides (Table 1). First, 10-fold serial dilutions (in water) of the two duplexes and their corresponding antisense strands were assayed in the qRT-PCR assay at room temperature (25°C) (Figure 1a). Comparison of the four amplification curves confirmed that modified and unmodified antisense strands were detected with equal efficiency, while less antisense strands were detected from the duplex under these same conditions. The underestimation of duplex concentrations ranged between 6-fold to 256-fold, and correlated with duplex concentration: at the highest concentrations, the average signal difference between duplex and single strand was 8 average cycle threshold units (AvCt), and at the lowest concentrations the difference was 2.5 Av Ct (Figure 1a). The difference in the detection of free antisense stands and the same antisense strands in the context of a duplex could be due to the inefficiency of RT in the presence of an equimolar amount of annealed sense strand found in the duplex samples. Comparison of the amplification curves of the modified and unmodified duplexes shows that the antisense strand from the unmodified duplex was slightly more efficiently detected than the antisense strand from the modified duplex. The decrease in RT efficacy for the chemically modified duplex is likely explained by its higher melting temperature (Table 1). Therefore, strand separation prior to RT may improve amplification curves for the detection of antisense strands from the siRNA duplex.

**Table 1:** Small interfering RNA (siRNA) duplexes

Text name	Alnylam name	Sequence	Melting temperature	Target
Duplex	AD1596			
Sense	Sense: A4639	GGAUCAUCUCAAGUCUUACdTdT	70.1°C	FVII
AS	AS: A4640	GUAAGACUUGAGAUGAUCCdTdT		Murine
				
Mod duplex	AD1661			
Mod sense	Sense: A4723	GGAUfCfAUfCfUfCfAAGUfCfUfUfACfdTsdT	83.4°C	FVII
Mod AS	AS: A4724	GUfAAGACfUfUfGAGAUfGAUfCfCfdTsdT		Murine
				
AD6490	AD6490			
A5296	Sense: A5296	GGAAUCuuAuAuuuGAUCcAsA	70.1°C	APOB
A5475	AS: A5475	uuGGAUcAAAuAuAAGAuUCcscsU		Human
				
AD1955	AD1955			
A3374	Sense: A3372	cuuAcGcuGAGuAcuucGAdTsdT	78.0°C	Luciferase
A5475	AS: A3374	UCGAAGuACUcAGCGuAAGdTsdT		Firefly

The sequences of the two duplexes AD1596 and AD1661 are identical, except that AD1661 contains chemical modifications. AD6490 and AD1955 are unrelated duplexes. The gene target of each siRNA is indicated. Chemical modifications are indicated as follows: 2'-O-Me-modified nucleotides are shown by lower case letters, 2'-fluoro-modified nucleotides are shown by 'f' following the modified nucleotide and phosphorothioate linkages are shown by 's'. All sequences are written in the 5'-3' direction.

Alnylam name refers to Alnylam Pharmaceuticals in-house name.

APOB = apolipoprotein B; AS = antisense; FVII = factor VII; mod = modified.

To test the effect of strand separation on siRNA detection by qRT-PCR, two siRNA duplexes and their matching antisense strands were heated for 10 minutes at 95°C and directly added into the RT reaction. Under these conditions, similar amplification curves were obtained for duplexes and single strands, particularly at concentrations equal or below 1.7 nmol siRNA per RT reaction (3.35 fmol per PCR reaction, see the -2.5 log siRNA point in Figure 1b), suggesting that duplex siRNAs can be accurately measured when the RT is performed on melted duplexes (Figure 1b).

*In vivo *delivery typically requires siRNAs formulation in LNPs prior to intravenous injection [11,13]. LNPs impose an additional barrier to quantification as the siRNA must be efficiently liberated from the particles before quantification. To test our ability to quantify LNP-formulated siRNAs, serial dilutions of the modified siRNA encapsulated in an LNP containing the novel lipid KC2 [5] (KC2 mod Dup), were compared to the modified unformulated siRNA (mod Dup) and the modified antisense strand (mod AS) (Figure 1c). As we had seen previously, unformulated duplex and single strands, heated at 95°C, showed similar amplification curves over at least four orders of magnitude, while formulated duplexes were amplified less efficiently. Therefore, we had to develop a method to disrupt LNP to liberate siRNAs for accurate quantification.

To release the siRNA from the LNP-KC2 formulation, we tested various detergents (data not shown) and ultimately selected 0.25% Triton X-100 for addition to the formulated siRNA prior to heating at 95°C. Under these conditions, nearly identical amplification curves were obtained for the modified antisense strand, modified duplex and LNP-KC2-formulated modified duplex (Figure 1d). A linear regression fit to the three amplification curves over five orders of magnitude showed an average slope of 3.2, which indicated nearly 100% efficient amplification under these modified conditions (Figure 1d).

**Figure 2:**
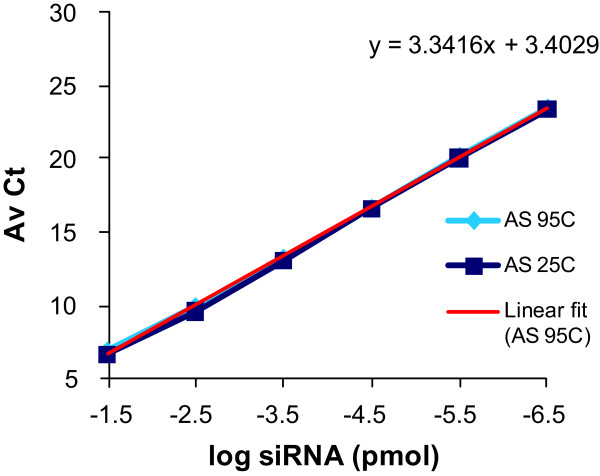
**Amplification curves of modified antisense (AS) strands**. AS strands were diluted in 0.25% Triton-phosphate-buffered saline (PBS), then were kept as indicated either at room temperature (25°C: AS 25C) or heated to 95°C for 10 min and added directly into reverse transcription reactions (AS 95C). The linear regression of the amplification curve for chemically modified AS at 95°C is shown (linear fit (AS 95C)). The average cycle threshold (AvCt) values indicate signals in single PCR reactions containing small interfering RNA (siRNA) equivalent concentrations ranging between 3.35 × 10^-2 ^to 3.35 × 10^-7 ^pmol. Each point is an average n = 3.

In order to confirm that the direct addition of the siRNA heated to 95°C in 0.25% Triton did not compromise reverse transcriptase activity, amplification curves of serially diluted antisense strands were evaluated with and without heating in triton. Similar amplification curves for the antisense strand under these two conditions confirmed that no enzyme inhibition had occurred (Figure 2). These results confirm that efficient amplification of antisense strands from LNP-formulated, chemically modified siRNA duplexes can be achieved under these conditions.

### High temperature is required to avoid reannealing

**Figure 3:**
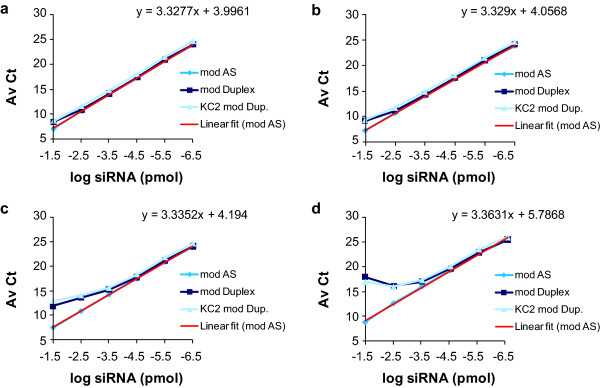
**Reannealing of the modified duplex**. The modified antisense (mod AS), modified duplex (mod Duplex) and lipid nanoparticle (LNP)-KC2-formulated duplex (KC2 mod Dup) small interfering RNAs (siRNAs) were diluted in 0.25% Triton-phosphate-buffered saline (PBS) and were first heated to 95°C for 10 min and then were kept for another 10 min at the indicated temperature before directly added into the RT reaction: **(a) **95°C, **(b) **85°C, **(c) **75°C, **(d) **25°C. The linear regression of the amplification curve for chemically modified AS is shown (linear fit (mod AS)). The average cycle threshold (AvCt) values indicate signals in single PCR reactions containing siRNA equivalent concentrations ranging between 3.35 × 10^-2 ^to 3.35 × 10^-7 ^pmol. Each point is an average n = 3.

The melting temperature (Tm) of the chemically modified duplex AD1661 was 83.4°C (Table 1). Based on this high Tm, it is expected that reannealing of melted duplex will occur rapidly upon cooling, thus explaining the reduced dynamic range of the assay (Figure 1b-d). To confirm this interpretation, modified antisense strand, modified duplex, and the LNP-KC2-formulated modified duplex were heated for 10 minutes at 95°C and were assayed either directly, or allowed to cool to 85°C, 75°C or 25°C for 10 minutes prior to RT (Figure 3). When assayed directly at 95°C, the amplification curves of all compounds were almost identical and linear over approximately 5 logs, with a slope of 3.3, which is characteristic of 100% efficiency (Figure 3a). However, careful analysis of the Ct values from inputs of >3.4E^-03 ^pmol (>-2.5 log siRNA) at 95°C, revealed an average of 1.4 Ct difference between modified antisense strand and the naked and formulated modified duplexes (Figures 1d and 3a). This difference can be explained by amplification inefficiencies observed when measuring highly concentrated duplexes that will more quickly anneal. The siRNA samples that were cooled down to 85°C were also efficiently amplified along most of the serial dilutions, except for the most concentrated duplex inputs that showed decreased amplification efficiencies of approximately 2 Av Ct units between the antisense strand and the tested duplexes (Figure 3b). Incubation at 75°C (Figure 3c) or 25°C (Figure 3d) resulted in more significant reductions in amplification efficiency for the duplexes at the highest concentrations tested, confirming that reannealing is both temperature and concentration dependent. These data show that complete and continued denaturation of the duplex at high concentrations is essential for optimal quantification.

### Quantification of modified and formulated siRNA from plasma and liver tissue

**Figure 4:**
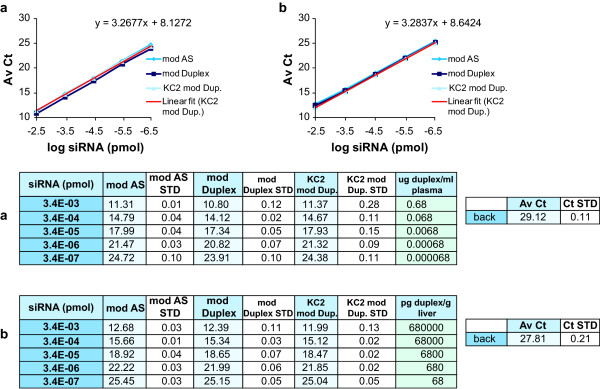
**Amplification curves of the modified antisense (mod AS), modified duplex (mod Duplex) and lipid nanoparticle (LNP)-KC2-formulated modified duplex (KC2 mod Duplex) in plasma and tissue**. The small interfering RNAs (siRNAs) were diluted in 0.25% Triton-phosphate-buffered saline (PBS) and **(a) **10% rat plasma or **(b) **100 mg/ml frozen powdered rat liver lysate. Reverse transcription reactions were made from heated siRNA dilutions at 95°C. The linear regression of the amplification curves of formulated KC2 mod Duplex are shown (linear fit (KC2 mod Dup)). The average cycle threshold (AvCt) values indicate signals in single PCR reactions containing siRNA equivalent concentrations ranging between 3.35 × 10^-3 ^to 3.35 × 10^-7 ^pmol. Given the final plasma and liver tissue concentrations, the standard curves reveal dynamic ranges of at least four orders of magnitude: 0.7 μg to 70 pg siRNA per 1 ml of plasma or 1 g of liver tissue. Background Ct values (back) are indicated. Each point is an average n = 3.

Having defined the conditions for efficient amplification of encapsulated chemically modified duplex siRNA in buffer, we next evaluated these conditions for siRNA in plasma and liver tissue. To do so, serial dilutions of LNP-KC2-formulated modified duplex, modified unformulated duplex, and modified antisense strand control were spiked into either naïve rat plasma (diluted 1:10 in 0.25% Triton heated to 95°C) or liver lysate (diluted to 100 mg/ml tissue in 0.25% Triton heated to 95°C). Spiked samples were centrifuged to remove debris and then reheated for 10 minutes at 95°C before direct addition into the qRT-PCR reaction. Nearly identical amplification curves were observed when comparing antisense strand alone with modified duplex and LNP-formulated modified duplex assayed in plasma (Figure 4a). Similarly, nearly indistinguishable amplification curves were observed when these same siRNAs were assayed in liver tissue (Figure 4b). The assays were linear over four orders of magnitude and the amplification curves had slopes of approximately 3.2, demonstrating efficient amplification. Given the final plasma and liver tissue concentration in those standard curves, we concluded that the dynamic ranges of the two assays were 0.7 μg to 70 pg siRNA per 1 ml of plasma and 0.7 μg to 70 pg of siRNA per 1 g of liver tissue (Figure 4).

**Figure 5:**
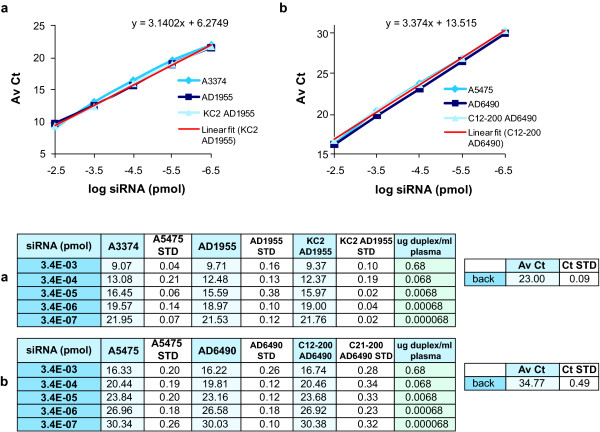
**Further examples of amplification curves of modified antisense (AS), modified duplex, and formulated modified duplex in plasma for the indicated small interfering RNAs (siRNAs)**. The modified siRNAs **(a) **AD1955 or **(b) **AD6490 were diluted in 0.25% Triton-phosphate-buffered saline (PBS)/10% rat plasma. Reverse transcription reactions were made from heated siRNA dilutions at 95°C. The linear regressions of the amplification curves of formulated KC2 or C12-200 modified duplexes are shown (linear fit (KC2 AD1955 or C12-200 AD6490)). The average cycle threshold (AvCt) values indicate signals in single PCR reactions containing siRNA equivalent concentrations ranging between 3.35 × 10^-3 ^to 3.35 × 10^-7 ^pmol. Background Ct values (back) are indicated. Each point is an average n = 3.

**Figure 6:**
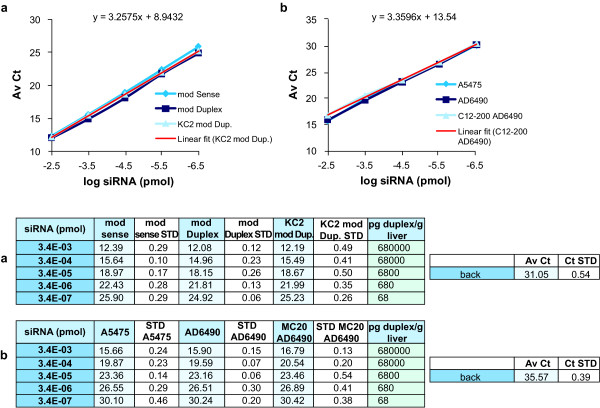
**Further examples of amplification curves of modified antisense (AS), modified duplex, and formulated modified duplex in liver tissue for the indicated small interfering RNAs (siRNAs)**. The modified siRNAs **(a) **AD1661 or **(b) **AD6490 were diluted in 0.25% Triton-phosphate-buffered saline (PBS) in rat liver at final concentration of 100 mg/ml. Reverse transcription reactions were made from heated siRNA dilutions at 95°C. For AD1661 the assay analysed the sense strand. In this experiment the modified sense strand A4723 serves as the single strand control. The linear regressions of the amplification curves of formulated KC2 or C12-200 modified duplexes are shown (linear fit (KC2 AD1661 or C12-200 AD6490)). The average cycle threshold (AvCt) values indicate signals in single PCR reactions containing siRNA equivalent concentrations ranging between 3.35 × 10^-3 ^to 3.35 × 10^-7 ^pmol. Background Ct values (back) are indicated. Each point is an average n = 3.

To confirm that our method could be used for the quantification of siRNAs containing chemically modified bases other than 2'-fluoro and formulations other than LNP-KC2, we tested two additional chemically modified and formulated siRNAs in plasma. AD1955 is a 2'-O-Me-modified siRNA formulated in LNP-KC2, while AD6490 is a 2'-O-Me-modified siRNA (Table 1), encapsulated in a distinct LNP formulation containing the lipid C12-200 (LNP-C12-200) [14]. Chemically modified antisense strands, duplexes and formulated duplexes were serially diluted in rat plasma and processed as described above. Similar amplification curves of the antisense strands, duplexes and formulated duplexes demonstrated a linear detection between 0.7 μg to 70 pg duplex/ml of plasma for both siRNAs (Figure 5). To further confirm the universal utility of this detection method, two additional siRNA spiking experiments were performed in rat liver lysates. In the first we used the 2'-fluoro-modified AD1661 duplex (Table 1) in the LNP-KC2 formulation, but this time, we tested reagents for the quantification of the sense strand (mod sense; Figure 6a). In the second experiment we tested the antisense strand, duplex and formulated AD6490 (Table 1) in LNP-C12-200 formulation (Figure 6b). Both experiments demonstrated a linear range over 4 logs and the ability to quantify siRNA between the ranges of 0.7 μg/g to 70 pg/g of liver tissue.

### Quantification of siRNA and target expression in rat liver

**Figure 7:**
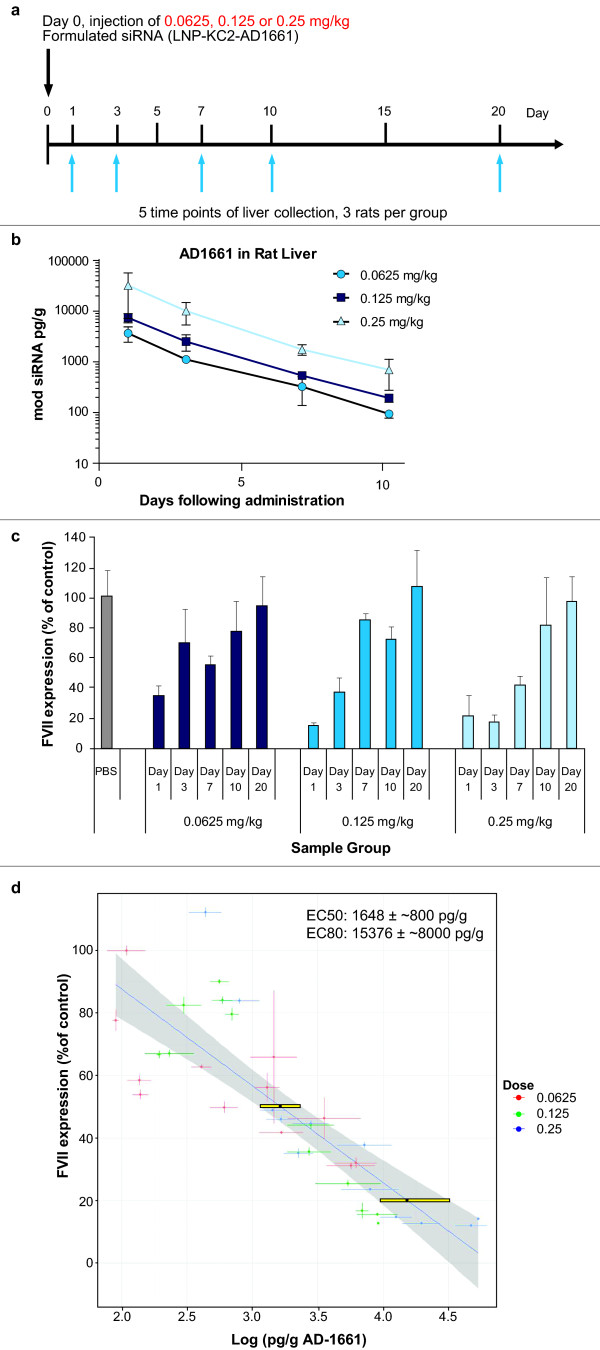
**Correlation between *in vivo *delivered small interfering RNA (siRNA) and target knockdown in rat liver**. **(a) **Experimental setup. Rats were tail-vein injected with phosphate-buffered saline (PBS), 0.0625, 0.125 and 0.25 mg/kg chemically modified and formulated siRNA in lipid nanoparticle (LNP)-KC2 (LNP-KC2-AD1661) on day 0. At 1, 3, 7, 10 and 20 days after injection, frozen powdered liver from three rats from each group was analysed for siRNA concentration and for target (factor VII (FVII)) expression. **(b) **Quantification of modified siRNA in rat liver. Average of three rats, measured twice independently, with day 20 levels below lower limit detection and therefore not shown (lowest limit of detection (PBS) = 25.8 ± 4.6 pg/g). **(c) **Gene target knockdown in rat liver. The average of FVII RNA expression in three rats, relative to FVII expression in PBS injected rats. **(d) **Correlation between siRNA drug levels and FVII RNA levels over time in rat livers. Symbols represent means ± 1 standard error (SE) for individual rats. Blue line, linear regression; grey shading indicates 95% confidence interval. Yellow rectangles indicate 50% effective dose (EC50) and EC80 estimates.

To quantify siRNAs *in vivo*, an intravenous dosing study was performed targeting FVII in rat liver. Single doses (0.0625, 0.125, or 0.25 mg/kg) of LNP-KC2-formulated modified duplex AD1661 were delivered to rats and both FVII RNA levels and siRNA concentrations were monitored over 20 days (Figure 7a). The drug elimination curves showed dose proportional clearance with average residual siRNA of 0.01% at day 10 in rat liver (Figure 7b). At all doses tested, siRNAs could be detected up to day 10 post dosing. A lower limit of detection (LLOD) of 25.8 ± 4.6 pg/g was calculated from the phosphate-buffered saline (PBS)-treated rats.

FVII transcript levels measured in liver by qRT-PCR assay decreased by as much as 80% at 1 day post siRNA administration (Figure 7c) and were tightly correlated with siRNA levels across the three doses and at all time points tested (see Figure 7d; linear regression significant at *P *< 0.01). Independent of the dose delivered, only 1.6 ± 0.8 ng of siRNA per 1 g of liver was needed to achieve the ED_50 _of FVII gene silencing (Figure 7d). Assuming 1.5 × 10^8 ^cells/g liver tissue [15], the amount of siRNA at the ED_50 _is equivalent to approximately 500 siRNA molecules/liver cell. The linear regression also indicates that to achieve 80% target depletion, approximately 15 ± 8 ng siRNA/g liver is required. Such concentrations were attained only on day 1 in the rats that were injected with 0.125 and 0.25 mg/kg of AD1661 (Figure 7c).

### Prediction of specificity: recognition of siRNA metabolites

**Figure 8:**
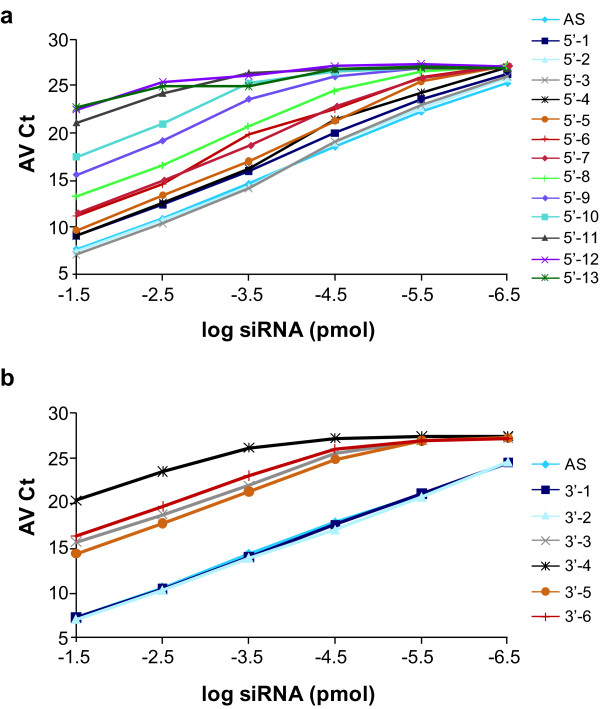
**The detection of full-length, 5' and 3' truncated antisense oligonucleotides**. **(a) **The 5'-truncated oligonucleotides (5' N-1 to 5' N-13) and **(b) **3'-truncated oligonucleotides (3' N-1 to 3' N-6) were tested in 10-fold serial dilutions by the heating-in-Triton quantitative reverse transcription PCR (HIT qRT-PCR) method. The sequences of these oligonucleotides and their nomenclature are detailed in Table 2. The average cycle threshold (AvCt) values indicate signals in single PCR reactions containing small interfering RNA (siRNA)-equivalent concentrations ranging between 3.35 × 10^-2 ^to 3.35 × 10^-7 ^pmol. Each point is an average n = 3.

The annealing of single strand RNAs to specific stem-loop RT oligonucleotides is a prerequisite for quantitative RT, which generates the first strand cDNA. Efficient synthesis of the second cDNA strand depends on the annealing of the newly synthesised cDNA to a specific forward primer. In this work, we designed stem-loop oligonucleotides that share 6 nucleotides of complementarity with the 3' end of the siRNA and forward primers with 18 nucleotides of complementarity with the 5' end of the first strand cDNA (see Methods). In addition to efficiently detecting full-length product, this primer design would be expected to allow detection of truncated siRNAs such as those generated though metabolism *in vivo*. To evaluate the degree of metabolite detection by this assay, a set of 19 truncated modified antisense strands were synthesised; 13 of these oligonucleotides contained 5' truncations and 6 contained 3' truncations (Table 2). We then tested the ability of the HIT qRT-PCR assay to discriminate among these truncated oligonucleotides. As expected based on the design features described above, we observed asymmetry between the ability to detect 5' and 3' truncated oligonucleotides (Figure 8). While there was a gradual loss in detection of 5' end truncations (Figure 8a), an abrupt loss of detection was observed with the elimination of only three bases on the 3' end of the antisense oligonucleotide (Figure 8b). Truncation of 6 nucleotides at the 5' end reduced the detection sensitivity by 10-fold compared to the full-length antisense strand and the truncation of 9 nucleotides reduced that signal by 100-fold (Figure 8a).

**Table 2:** Set of 5' and 3' truncations of the modified antisense A4724.

Truncation name	Sequence 5'-3'
Full-length AS	GUfAAGACfUfUfGAGAUfGAUfCfCfdTsdT
	
5' N-1	UfAAGACfUfUfGAGAUfGAUfCfCfdTsdT
5' N-2	AAGACfUfUfGAGAUfGAUfCfCfdTsdT
5' N-3	AGACfUfUfGAGAUfGAUfCfCfdTsdT
5' N-4	GACfUfUfGAGAUfGAUfCfCfdTsdT
5' N-5	ACfUfUfGAGAUfGAUfCfCfdTsdT
5' N-6	CfUfUfGAGAUfGAUfCfCfdTsdT
5' N-7	UfUfGAGAUfGAUfCfCfdTsdT
5' N-8	UfGAGAUfGAUfCfCfdTsdT
5' N-9	GAGAUfGAUfCfCfdTsdT
5' N-10	AGAUfGAUfCfCfdTsdT
5' N-11	GAUfGAUfCfCfdTsdT
5' N-12	AUfGAUfCfCfdTsdT
5' N-13	UfGAUfCfCfdTsdT
	
3' N-1	GUfAAGACfUfUfGAGAUfGAUfCfCfdT
3' N-2	GUfAAGACfUfUfGAGAUfGAUfCfCf
3' N-3	GUfAAGACfUfUfGAGAUfGAUfCf
3' N-4	GUfAAGACfUfUfGAGAUfGAUf
3' N-5	GUfAAGACfUfUfGAGAUfGA
3' N-6	GUfAAGACfUfUfGAGAUfG

The full-length antisense (AS) strand sequence is indicated, along with the sequences of 5' (5' N-1 to 5' N-13) and 3' (3' N-1 to 3' N-6) truncated oligonucleotides. Chemical modifications are shown as follows: 2'-fluoro-modified nucleotides are shown by 'f' following the modified nucleotide and phosphorothioate linkages are shown by 's'. All sequences are written in the 5'-3' direction.

## Discussion

**Figure 9:**
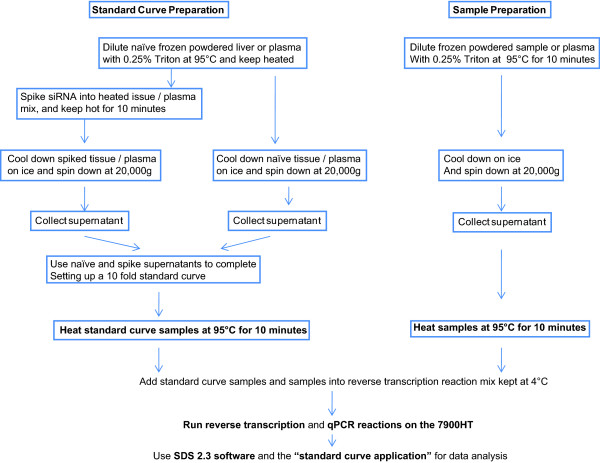
**Schematic presentation of standard curve and sample preparation protocols for the heating-in-Triton (HIT) quantification of small interfering RNA (siRNA) from plasma and liver tissue**.

The HIT qRT-PCR assay (Figure 9) represents a significant improvement over the previously published stem-loop qRT-PCR method [8] as it allows a reliable and sensitive quantification of the sense or antisense strand of formulated and chemically modified siRNAs from *in vivo *samples. The improvements include protocols for processing tissue samples without the need for RNA purification. The resulting assay has a dynamic range exceeding four orders of magnitude and a sensitivity that surpasses other published methods [4,7,9]. For plasma and tissue detection, samples are directly diluted in 0.25% Triton and heated to 95°C to efficiently release the encapsulated siRNA from the LNP. To achieve efficient amplification, plasma and tissue samples are then centrifuged to remove debris, and reheated to 95°C to ensure efficient strand separation for the RT reaction. Comparable quantitation of antisense strands alone versus modified and formulated duplexes confirms that complete duplex denaturation occurs.

The HIT siRNA quantification method extends the work of Chen *et al*., who developed the original stem-loop qRT-PCR method for the quantification of miRNAs [8]. They demonstrated high assay sensitivity and showed its broad utility by measuring the levels of 10 different miRNAs from tissue culture cells, using various sample preparation techniques. Specifically, they observed that quantification from a PBS-boiled tissue culture suspension was as efficient as that from purified RNA, thus eliminating the RNA purification step in their protocol. While the direct boiling/cooling method described by Chen was simple, it was clear that additional steps were needed to make this protocol suitable for the quantification of chemically modified and formulated siRNAs from animal tissue. Importantly, efficient strand separation turned out to be critical for accurate quantification of siRNAs by qRT-PCR. Strand reannealing occurs rapidly in cooled siRNA lysates. As a result, the direct application of heat-denatured siRNAs into the RT reaction was required when measuring high concentrations of high Tm duplexes such as AD1661. In addition, effective siRNA quantification required complete release of the siRNA from the liposomal formulation, a challenge not relevant to endogenous miRNA detection assays. To accomplish this, 0.25% Triton at 95°C was added, as heating in PBS alone was not sufficient to fully liberate the duplex.

The rate of duplex reannealing is directly correlated with duplex concentration and inversely correlated with increased temperature. The highest siRNA concentration in an RT reaction used in our experiments was 17 nmol, from which 33.5 fmol were analysed in one qRT-PCR reaction. At that concentration, reannealing of duplexes occurred rapidly and amplification was not fully efficient, as evidenced by the non-linearity of the curves at this input dose (see the -1.5 points in Figures 1d and 3a). Based on this observation, the upper limit of quantification for our standard curves was defined as 3.4 fmol per qRT-PCR reaction. This translated to approximately 0.7 μg duplex/ml plasma and 0.7 μg duplex/g of liver tissue (Figure 4). The average lowest limit of quantification was 0.34 attmol of siRNA, translated to approximately 70 pg duplex/ml plasma and 70 pg duplex/g of liver tissue, yielding signals that are 4-fold to 10-fold above background signal (a difference of 2-3 Av Ct units).

Having developed an assay for the quantification of formulated and chemically modified siRNA in liver tissue or plasma, the next challenge was to use this assay for the quantification of such siRNAs delivered to rats by intravenous dosing. The goal was to quantify the siRNA antisense strand from siRNA duplex that targets FVII mRNA in rat liver. The antisense strand was selected as it represents the functional targeting strand, but the assay is equally amenable to sense strand detection (Figure 6a). In this *in vivo *experiment, 0.0625, 0.125 and 0.25 mg/kg of LNP-KC2-formulated AD1661 were delivered as single doses, and samples were quantified for both target mRNA and siRNA levels at 1, 3, 7, 10, or 20 days post dosing. A linear correlation between the amounts of siRNA delivered and target knockdown was observed in the rat livers (Figure 7). This correlation indicated that independent of the dose administered, approximately 1.6 ± 0.8 ng/g of modified AD1661 siRNA resulted in an approximately 50% reduction of FVII mRNA (ED_50 _= approximately 1.6 ± 0.8 ng/g). This translates to an average of 500 siRNA molecules/cell in rat liver.

To confirm that extraction of siRNA using the HIT qRT-PCR protocol is efficient, we repeated the assay on a subset of the above rat liver tissue samples using a phenol chloroform extraction protocol. The Ct values measured from these two assay protocols were almost identical suggesting that the HIT qRT-PCR assay protocol efficiently liberates siRNAs for easy detection (data not shown). This experiment not only confirmed the quantification results by another method, but also assured us that our extraction method results in efficient release of protein bound siRNA, such as might be bound to the Argonaute 2 (Ago2) protein.

By analysing sets of truncated antisense molecules we aimed to predict the type of siRNA metabolites that one may detect when using the HIT qRT-PCR method. As expected, the assay is more sensitive to 3' end truncations than to 5' end truncations (Figure 8). We observed that siRNA metabolites that include strand truncations of up to five nucleotides at their 5' end and up to two nucleotides on their 3' end are detected by the HIT qRT-PCR method with reasonable efficiency. Future assay development, including modifications of primer length, will further improve HIT qRT-PCR's ability to specifically measure full-length and siRNA metabolites.

## Conclusions

In summary, we developed a reliable, reproducible and highly sensitive method of quantitating siRNAs delivered *in vivo*. As more siRNA therapeutic programmes are initiated and move into preclinical and clinical stages, accurate quantification of siRNA levels across various tissues will become crucially important. This method allows the correlation of pharmacodynamic with precise pharmacokinetic measures, using a sensitive and easy to perform assay that will contribute to the successful development of siRNA therapeutics.

## Methods

Oligonucleotides (Integrated DNA Technologies, IA, USA) and TaqMan probes (Applied Biosystems of Life Technologies, CA, USA) (Table 3) were designed according to Chen *et al*. [8].

**Table 3:** Oligonucleotides and TaqMan probes for the stem-loop real time PCR assays.

Assay	Primer/probe	Sequence
AD1596 AD1661	Stem-loop RT	GTCGTATCCAGTGCAGGGTCCGAGGTATTCGCACTGGATACGACAAGGAT
	Forward primer	GCCCGCGTAAGACTTGAGATGATC
	Reverse primer	GTGCAGGGTCCGAGGT
	TaqMan probe	(6-FAM) CTGGATACGACAAGGAT (MGB)
		
AD6490	Stem-loop RT	GTCGTATCCAGTGCAGGGTCCGAGGTATTCGCACTGGATACGACAGGGAA
	Forward primer	CCCGCTTGGATCAAATATAAGATT
	Reverse primer	GTGCAGGGTCCGAGGT
	TaqMan probe	(6-FAM) CTGGATACGACAGGGAA (MGB)
		
AD1955	Stem-loop RT	GTCGTATCCAGTGCAGGGTCCGAGGTATTCGCACTGGATACGACAACTTA
	Forward primer	TCGTGTTCGAAGTACTCAGCGTAA
	Reverse primer	GTGCAGGGTCCGAGGT
	TaqMan probe	(6-FAM) CTGGATACGACAACTTA (MGB)
		
AD1661 (sense)	Stem-loop RT	GTCGTATCCAGTGCAGGGTCCGAGGTATTCGCACTGGATACGACAAGTAA
	Forward primer	GCCCGCGGATCATCTCAAGTCTTA
	Reverse primer	GTGCAGGGTCCGAGGT
	TaqMan probe	(6-FAM) CTGGATACGACAAGTAA (MGB)

All assays designed to detect the antisense strands of the indicated duplexes, except for the indicated assay designed for sense strand detection. All sequences are written in the 5'-3' direction.

6-FAM = 6-carboxyfluorescein; MGB = minor groove binder; RT = reverse transcription.

siRNAs were formulated either in LNP-KC2 [5], or in LNP- C12-200 [14].

### Quantification of siRNA in liver tissue or plasma in 0.25% Triton: the HIT qRT-PCR protocol (Figure 9)

Following plasma collection and necropsy, plasma and liver samples were kept frozen and liver tissue ground for 2 minutes (min) at 250 strokes/min using a Geno Grinder (SPEXP SamplePrep, NJ, USA) to achieve a light homogeneous powder.

### Preparation of standard curves

Duplex, antisense and formulated siRNAs (20 μM) were used for the preparation of serial 10-fold dilutions into 95°C boiled tissue (100 mg/ml) or plasma (1:10 diluted) in 0.25% Triton X-100 in PBS, as detailed below.

For tissue, 500 μl of 0.25% Triton X-100 at 95°C was added directly into 50 mg of naïve (PBS-treated animal) frozen powdered tissue. The lysate was vortexed and put back into the 95°C hot block. Then, 12.5 μl of 20 μM siRNA was added into the hot lysate, which was then vortexed again and put back into the hot block for a total incubation time of 10 min. This procedure was performed once for formulated duplex, naked duplex and for antisense or sense strands. Additional tubes with naïve tissue lysates in 0.25% Triton X-100 were heated at 95°C. These were used for the preparation of the standard curve points. Following 10 min at 95°C, all lysates were cooled on ice for 10 min.

For plasma, plasma from naïve animals (PBS treated) was diluted 1:10 in 0.25% Triton X-100, and 500 μl taken into an Eppendorf tube and heated in a 95°C hot block. Then, 12.5 μl of 20 μM siRNA was added into the hot lysate, which was then vortexed again and put back into the hot block for a total incubation time of 10 min. This procedure was performed once for formulated duplex, naked duplex and for antisense or sense strands. Additional tubes with naïve plasma lysates in 0.25% Triton X-100 were heated at 95°C. These were used for the preparation of the standard curve points. Following 10 min at 95°C, all lysates were cooled on ice for 10 min.

Cooled tissue and plasma lysate tubes were centrifuged at 20,000 *g *for 20 min at 4°C and supernatants placed into clean Eppendorf tubes and kept on ice.

The naïve and siRNA-spiked (500 nmol), heated and cleared tissue or plasma lysates were used to prepare 10-fold serial dilution points (50 nmol to 50 fmol) for the standard curves.

### Preparation of liver and plasma samples for the quantification of siRNA

For tissue, 500 μl of 0.25% Triton X-100 at 95°C was added directly into each 50 mg frozen powdered tissue sample. Lysates were vortexed and put back into the 95°C hot block for a total incubation time of 10 min. Lysates were vortexed twice more during this incubation. Following 10 min at 95°C, all lysates were cooled on ice for 10 min.

For plasma, plasma samples were diluted 1:10 in 0.25% Triton X-100. Then, 500 μl from each diluted plasma sample was heated at 95°C for a total incubation time of 10 min and vortexed twice during this incubation. Following 10 min at 95°C, all lysates were cooled on ice for 10 min.

All tissue and plasma lysates were centrifuged at 20,000 *g *for 20 min at 4°C and supernatants taken into clean Eppendorf tubes and kept on ice.

### Reverse transcription reactions

Reverse transcription reactions were performed using a TaqMan MicroRNA Reverse Transcription kit 200 (Applied Biosystems of Life Technologies, cat # 4366596).

We recommend using two adjacent PCR machines for the procedure. One PCR machine for heating the 'boiling plate' and the second for the 'RT plate', as detailed below.

A total of 50 μl from each standard curve point (single strand, duplex and formulated duplex), one naïve (for background) and sample lysates were aliquoted into one PCR plate ('boiling plate') and heated at 95°C for 10 min on one PCR machine. Then, 10 μl of RT reaction mix (100 mmol deoxyribonucleotide triphosphates, 250 nmol stem and loop oligonucleotides, 20 U/μl RNase inhibitor, 1 × RT buffer, 50 U/μl MultiScribe Reverse Transcriptase) was aliquoted into each well of the 'RT plate', which was placed on the second adjacent PCR machine and kept at 4°C. Following 10 min of heating, the cover from the 'boiling plate' was removed and, while the plate was kept on the heating block, 5 μl from each hot sample was directly added into the RT reaction mix (at 4°C) on the 'RT plate' and the program was switched onto the RT program (30 min, 16°C, 30 min, 42°C, 5 min, 85°C).

### PCR amplification

A total of 2 μl of cDNA from the previous step was added into the PCR amplification reaction mix (0.2 μM TaqMan probe, 1.5 μM forward primer, 0.7 μM reverse primer, TaqMan 2 × Universal PCR Master Mix, No AmpErase UNG; Applied Biosystems of Life Technologies, cat # 4366596). The ABI 7900HT Sequence Detection System and the 'Standard Curve' application SDS 2.3 (Applied Biosystems of Life Technologies) were used to run the PCR reaction.

### Quantification of Gene knockdown in rat liver

An RNeasy mini kit (Qiagen Inc. Valencia, CA, USA, cat # 74106) was used to purify total RNA from frozen powdered rat liver. A High Capacity cDNA Reverse Transcription Kit (Applied Biosystems of Life Technologies, cat # 4368814) was used for RT. TaqMan Assays Rat FVII (cat # Rn00596104_m1) and TaqMan Assay Rat GAPDH kit (cat # 4352338E) together with TaqMan Universal PCR Master Mix No AmpErase UNG (cat # 4324020, all from Applied Biosystems of Life Technologies) were used for the amplification of rat FVII and GAPDH transcripts.

## Competing interests

The authors declare that they have no competing interests.

## Authors' contributions

YL conceived the study, designed and performed experiments, analysed results, and drafted the manuscript. NS and AC performed experiments and analysed results. XZ performed the animal experiments, BRB performed statistical analysis SK and KD designed and synthesised the truncated oligonucleotides, SS performed Tm measurements. MG performed initial experiments and analysed results. AA planned the *in vivo *experiment and with RH provided critical analysis of the *in vivo *data. RM provided critical review and discussion and manuscript revision. All authors read and approved the final manuscript.
